# Cut‐off points for serum ferritin to identify low iron stores during the first year of life in a cohort of Mexican infants

**DOI:** 10.1111/mcn.13205

**Published:** 2021-05-25

**Authors:** Azucena Pérez‐Acosta, Ximena Duque, Belem Trejo‐Valdivia, Samuel Flores‐Huerta, Sergio Flores‐Hernández, Gloria Martínez‐Andrade, Marco González‐Unzaga, Bernardo Turnbull, Ericka Escalante‐Izeta, Miguel Klünder‐klünder, Tania Carranco‐Hernández, Eugenia Mendoza, Elma Ivonne Sotelo‐Ham, Alicia Pineda, Carolina Medina‐Escobedo, Homero Martinez

**Affiliations:** ^1^ Unidad de Investigación Médica en Enfermedades Infecciosas y Parasitarias, Centro Médico Nacional Siglo XXI Instituto Mexicano del Seguro Social Ciudad de México México; ^2^ Centro de Investigación en Nutrición y Salud Instituto Nacional de Salud Pública Cuernavaca Morelos Mexico; ^3^ Unidad de Investigación en Salud Comunitaria, Hospital Infantil de México Federico Gómez Instituto Nacional de Salud Ciudad de México México; ^4^ Centro de Investigación en Evaluación y Encuestas Instituto Nacional de Salud Pública Cuernavaca Morelos Mexico; ^5^ Unidad de Investigación Epidemiológica y en Servicios de Salud Instituto Mexicano del Seguro Social Ciudad de México México; ^6^ Unidad de Investigación en Epidemiología Nutricional Instituto Mexicano del Seguro Social Ciudad de México México; ^7^ Académico de tiempo Universidad Iberoamericana Ciudad de México México; ^8^ Departamento de Ciencias de la Salud Universidad Iberoamericana San Andrés Cholula Puebla Mexico; ^9^ Subdirección de Investigación, Hospital Infantil de México Federico Gómez Instituto Nacional de Salud Ciudad de México México; ^10^ Dirección de Prestaciones Económicas y Sociales, Coordinación del Servicio de Guardería para el Desarrollo Integral Infantil, División de Desarrollo Integral Infantil Instituto Mexicano del Seguro Social Ciudad de México México; ^11^ Coordinación Delegacional de Investigación de Zacatecas Instituto Mexicano del Seguro Social Zacatecas México; ^12^ Unidad de Investigación en Epidemiología Clínica Instituto Mexicano del Seguro Social Colima México; ^13^ Dirección de Educación e Investigación en Salud. Unidad Médica de Alta Especialidad, Hospital de Especialidades Centro Médico Nacional “Ignacio García Téllez” en Mérida Instituto Mexicano del Seguro Social Mérida México; ^14^ Dirección de Investigación, Hospital Infantil de México Federico Gómez Instituto Nacional de Salud Ciudad de México México; ^15^ Global Technical Services Nutrition International Ottawa Ontario Canada

**Keywords:** assessment of iron status, nutrition, epidemiology, infant iron status, iron deficiency, *K*‐means cluster analysis, low iron stores, public health, serum ferritin, serum ferritin cut‐off points

## Abstract

The aim of this study was to identify serum ferritin (SF) cut‐off points (COPs) in a cohort of healthy full‐term normal birth weight infants who had repeated measurements of SF and haemoglobin every 3 months during the first year of life. The study included 746 full‐term infants with birth weight ≥2,500 g, having uncomplicated gestations and births. Participants received prophylactic iron supplementation (1 mg/day of iron element) from the first to the 12th month of life and did not develop anaemia during the first year of life.

Two statistical methods were considered to identify COPs for low iron stores at 3, 6, 9 and 12 months of age: *deviation from mean* and *cluster analysis*. According to the *K‐means cluster analysis* results by age and sex, COPs at 3 and 6 months for girls were 39 and 21 μg/L and for boys 23 and 11 μg/L, respectively. A single COP of 10 μg/L was identified, for girls and boys, at both 9 and 12 months. Given the physiological changes in SF concentration during the first year of life, our study identified dynamic COPs, which differed by sex in the first semester. Adequate SF COPs are necessary to identify low iron stores at an early stage of iron deficiency, which represents one of the most widespread public health problems around the world, particularly in low‐ and middle‐income countries.

**Abbreviations**
95% CI: 95% confidence interval
COP: cut‐off point
CV: coefficient of variation
EB: exclusive breastfeeding
Hb: haemoglobin
ID: iron deficiency
IDA: iron deficiency anaemia
IMSS: Mexican Institute for Social Security
SF: serum ferritin
WHO: World Health Organization

**Key messages**

- The most common iron biomarker used to identify iron deficiency is serum ferritin (SF). However, there is no consensus about the cut‐off points for SF to identify low iron stores in infants. The cut‐off limits currently used were generated from data from older children that were extrapolated to infants.
- Major physiological changes in ferritin concentration occur during the first year of life; for this reason, using only one cut‐off value to define low iron stores throughout infancy is not appropriate. In this study, SF concentration showed a marked decrease from 3 to 6 months; it was lower from 6 to 9 months and remained with no significant change from nine to 12 months of life.
- SF concentration was higher in girls than in boys at 3, 6, and 9 months of life but was similar at 12 months. In this study, we identified dynamic COPs for SF concentration using a *K‐means cluster analysis* approach based on the distribution of SF by age and sex, to define low iron stores that differed by sex in the first semester.
- It is important to identify ID before the onset of IDA. IDA is the last stage of a process of deterioration of iron status, a stage in which the production and concentration of haemoglobin is lower than what is considered normal.

## 1 INTRODUCTION

Iron is needed for neuronal myelination, neurogenesis and differentiation of neurites and is indispensable for the adequate function of all tissues. In infants, adequate nutritional iron status depends on different factors such as length of gestation, birth weight, time of clamping of the umbilical cord and mother's iron stores, among others (Domellöf et al., 2014; Hernell et al., 2015). Under normal conditions during gestation and birth, and with the dietary offering of iron from breast milk during the first 6 months of life, it is expected that iron nutritional status would be adequate during this period (Black, 2012). From 6 to 24 months of life, meeting the recommended daily intake of iron just by the diet is often a challenge. Between 6 and 24 months of age, infants are highly prone to iron deficiency (ID), especially in low‐ and middle‐income countries, because their diets offer less quantity and lower quality iron than that required (Lönnerdal et al., 2015; WHO, 2017). To cover the high requirements demanded by this period of active growth and tissue accretion, it is necessary to continue with breast milk, complemented by iron‐rich foods, including foods of animal origin as well as legumes, vegetables, fruits and iron‐fortified cereals (Aggett et al., 2002; Dewey, 2013; Hernell et al., 2015).

Serum ferritin (SF) concentration is the most widely used biochemical indicator to assess ID (Garcia‐Casal et al., 2018; WHO, 2007, 2011). Reduced iron stores limit the production of haemoglobin (Hb), eventually leading to iron deficiency anaemia (IDA) (Domellöf, et al., 2014; Hernell et al., 2015). Anaemia is identified by a haemoglobin level below recommended cut‐offs, specific by age, sex, physiological status and altitude above sea level. There are many causes of nutritional anaemia, including deficiencies of micronutrients like vitamins A, B_2_, B_6_, B_9_, B_12_, C, D and E, or minerals, including iron, copper and zinc. Other non‐nutritional factors may also cause anaemia, for example, in response to inflammatory or infectious processes (WHO, 2020). Therefore, by only measuring haemoglobin, it is not possible to identify the cause of anaemia.

ID or IDA is the most prevalent nutritional deficiency in the world (Gupta et al., 2016; WHO, 2015). In the United States, according to data from the National Health and Nutrition Examination Survey of 2007–2010, the prevalence of ID in children 1–2 years of age was 13.5% (95%CI: 9.8–17.2), 5.4% (95% CI: 3.5–7.4) of anaemia and 2.7% (95% CI: 1.2–4.2) of IDA (Gupta et al., 2016). In Mexico, results from the 2006 National Health and Nutrition Survey show that the prevalence of anaemia in children 1–4.9 years was 20.4%. Anaemia was associated with ID in 42.2% of cases (95% CI: 32.3–52.6) (De la Cruz‐Góngora et al., 2012). And in 2016, the prevalence of anaemia reported by Halfway National Health and Nutrition Survey in Mexico in children 1–4.9 years was 26.9% (95% CI: 23.3–30.9); the prevalence was higher in the group of children 1–1.9 years, 37.9% (95% CI: 29.9–46.7) (De la Cruz‐Góngora et al., 2018). In Mexico in 2002, a study with a representative sample of children less than 2 years of age (beneficiaries of health care services by the Mexican Institute for Social Security [IMSS]) showed that in urban infants less than 6 months old, 4.7% had ID and 9.8% had anaemia (1.9% had IDA and 7.9% had anaemia not related to ID). In infants between 6 and 11 months of age, 18.9% had ID and 21.0% had anaemia (7.5% had IDA and 13.5% had anaemia not related to ID) (Duque et al., 2007). There is little information about anaemia and ID prevalence in children under 1 year of age.

The concentration of SF throughout the first year of life tends to decrease as a reflection of the use of iron reserves during this phase of accelerated growth and development. In healthy children in Zimbabwe, Miller reported a geometric mean of SF at birth of 199 μg/L, 52 μg/L at 3 months, and 15 μg/L at 6 months (Miller et al., 2006). Reinbott found that the geometric mean of SF in healthy children in Cambodia decreased significantly between 3 and 6 months, with a lower speed of decrease between 10 to 24 months: from 3 to 6 months, the geometric mean was 50 μg/L; from 6 to 12 months, it was 15 μg/L and from 12 to 24 months, it was 14 μg/L (Reinbott et al., 2016).

The SF cut‐off point (COP) recommended by the World Health Organization (WHO) to diagnose low iron stores for children less than 2 years of age is 10 or 12 μg/L (WHO, 2007, 2011). However, their application is limited in infants because these COPs were generated based on data from older children that were extrapolated to infants (Garcia‐Casal et al., 2018). There have been few studies that have described laboratory reference intervals for the assessment of iron status in young children, but the information pertaining to the first 12 months of life is very limited and does not fully describe the dynamic changes found in iron status during this period (Colantonio et al., 2012; Larsson et al., 2019; Parkin et al., 2017).

Assessment of serum ferritin, with specific reference intervals for infants, can facilitate the identification of low iron stores at an early stage of ID if SF is below the lower limit. There is evidence that sub‐clinical iron deficiency (i.e., before haemoglobin synthesis is affected) may have long‐term consequences affecting behaviour and neurodevelopment (Lozoff et al., 2000). Given that SF values are higher in the first months of life but decrease rapidly with age, a single COP does not seem adequate to evaluate ID over the first year of life. However, there is controversy over which values are adequate for infants, given the physiological changes found in this age group. Based on a study of healthy children in Honduras and Sweden using the geometric mean less two standard deviations (SD), Domellöf proposed the following reference values for SF for diagnosis of ID during the first year of life: 20 μg/L (at 4 months), 9 μg/L (at 6 months) and 5 μg/L (at 9 months) (Domellöf et al., 2001; Domellöf, Dewey, et al., 2002; WHO, 2007, 2011). Following a study with healthy Chinese children, Wu proposed a COP to identify low iron stores of 16 μg/L at 4 months and 11 μg/L at 6 months (Wu et al., 2016). The main objective of this study was to identify dynamic COPs for SF concentration to define low iron stores at specific ages during the first year of life in a cohort of Mexican infants.

## 2 PARTICIPANTS AND METHODS

We carried out a secondary analysis of information obtained from a multicentre randomized clinical trial that included prophylactic iron supplementation and a follow‐up between the first to the 12th month of life for a cohort of full‐term and clinically healthy infants with normal birth weight. The parent study was carried out between July 2002 and August 2005 (Martinez et al., 2008). Infants' parents gave their informed consent before their children were included in the study. The study was conducted following the Declaration of Helsinki, and the protocol was approved by the Ethics Committee of IMSS.

### 2.1 Inclusion criteria

Infants who received care services provided by IMSS were eligible to participate in the study. The parent study included clinically healthy full‐term infants (37–42 weeks of gestation), with a birth weight ≥2,500 g, whose mothers had a non‐complicated pregnancy and delivery. The participants were recruited in four cities around the country. Mothers who attended antenatal care visits were invited to participate. The infants entered the study when they were 30 ± 7 days old and were randomly assigned to prophylactic oral iron supplementation offered weekly (7 mg/dose of iron element as ferrous sulphate or aminochelate iron) or monthly (28 mg/dose of iron element as ferrous sulphate or aminochelate iron).

### 2.2 Explanatory variables

Parental socioeconomic information was obtained by interview (age, schooling and occupation), and socioeconomic status was calculated based on educational and occupational levels of one or two infants' parents for single or dual wage earners (Hollingshead, 1975). Clinical variables at birth were extracted from the clinical record. Information on infant's diet was collected monthly, using a food frequency questionnaire recording food consumption the previous month. Infants' complementary feeding from 6 months of age was evaluated with an adaptation of the index proposed by Ruel et al., which included information on breastfeeding; formula or bottle feeding; dietary diversity (consumption of food groups/week: cereals or legumes, tubers, dairy, egg or fish or poultry, red meat and others) and frequency of consumption (food groups/week). The index score has a range of 0–12 points, where a higher value indicates better quality of complementary diet. Also, the intake of iron‐rich or iron‐fortified foods was determined from 6 months of age by the consumption of meat, iron‐fortified commercial foods or iron‐fortified powder used at home (Ruel & Menon, 2002; WHO, 2008). Anthropometric measurements were also taken monthly, including weight and length; weight‐for‐age and length‐for‐age indicators were calculated using the WHO Child Growth Standards as a reference and expressed as Z scores (WHO, 2006).

### 2.3 Serum ferritin

From the third month of age and every 3 months thereafter, a venous blood sample was taken from the infants by an experienced phlebotomist. No more than two attempts were done to obtain the sample. The appointment was rescheduled if the infant showed any sign or symptom of respiratory or gastrointestinal disease or when the infant had been vaccinated the previous week. The blood sample was taken from a vein on the dorsum of the hand with safety‐multifly needle 20G (Sarstedt, Nümbrecht, Germany) collecting drops into two microtubes of 500 μl. One tube contained ethylenediamine tetraacetic acid (EDTA) for a complete blood count, which was measured by automated haematology analyser (DXH600 Coulter Cellular, Beckman Coulter Inc, CA, USA) on‐site in each participating clinic. The second microtube had separator gel to determine SF, which was centrifuged for 20 min at 3,000 rpm; the serum was aliquoted and frozen at −20°C in cryovials in each clinic and then transported on iceboxes at 4–5°C to a central location, in Mexico City, where the samples were kept at −70°C until analysis. The determination of SF was made by the immuno radioanalysis method (Immunotech‐IRMA, IM 3492, Praha, Czech Republic) in the clinical laboratory of the Pediatric Hospital of the Century XXI Medical Center, IMSS, following the laboratory standard operating procedures. According to the assay manufacturer, the traceability methodology for the values assigned to calibrators and control materials in this kit was in accordance with the 3rd International Standard (NIBSC; recombinant ferritin; 94/572). This kit has five calibrators with concentrations ranging from 0 to 1,000 μg/L and two controls with concentrations of 45.6–72.8 μg/L and 177–283 μg/L. The performance of the method was evaluated by the degree of uncertainty reported as the intra‐inter‐assay coefficient of variation (CV) of the components of the standard curve (calibrators, % maximum binding, % non‐specific binding and ED 20%, 50%, and 80%) and controls in 43 assays which were less than 20%, which is among the ranges allowed for radiometric assays.

Infants who did not develop anaemia during the year of follow‐up (*n* = 746) were included in the present analysis. Infants who presented anaemia (*n* = 239) were treated with oral iron and were excluded from this analysis. This exclusion was required due to the objective of this study, that is, to identify SF COPs to define low iron stores, the first stage of ID, when haemoglobin levels are still normal. Anaemia was defined using haemoglobin adjusted for altitude and the specific cut‐off for age (Hb < 95 g/L at 3 months, <105 g/L at 6 and 9 months and <107 g/L at 12 months) (Duque et al., 2007). Given that infants were not always brought to their appointments on the scheduled day for the blood sample draw and given the need to identify SF concentration on specific ages, we only included those infants whose blood samples were taken at the expected ages according to the following criteria: 90 ± 15 days for 3 months, 180 ± 15 days for 6 months, 270 ± 15 days for 9 months and 365 ± 15 days for 12 months.

### 2.4 Statistical analyses

Given that the distribution of SF is skewed to the right, geometric means and 95% CI by periods of measurement (90 ± 15 days, 180 ± 15 days, 270 ± 15 days and 365 ± 15 days of age) were used to describe the data. For each period, outlier values (±3 SD around the mean) were removed. Kernel density estimators were used to analyse SF distribution.

Graphics of SF distribution and statistical comparisons of the geometric means and medians of SF (by period of measurement) were analysed to evaluate possible differences in SF distribution due to explanatory variables (sex, gestational age, type of birth, birth weight, socioeconomic level and frequency of prophylactic supplementation) using density, probability distribution and scatter plots, Kolmogorov–Smirnov, Student *T* test, Kruskal–Wallis and Mann–Whitney *U* tests, as appropriated. SF concentration showed differences by sex at 3, 6, and 9 months. No other significant differences were found. According to these results, the analysis to identify the COPs were carried out separately by period of measurement and stratified by sex.

Two approaches were used to identify SF COPs to assess low iron stores: (1) the *deviation from mean* approach, which identifies the extreme values as large deviations from the mean under a normal distribution assumption, and (2) *K‐means clustering* to form relative groups of individuals (or values) based on a specific statistical criterion. The extreme values related to the first approach were obtained for the log‐SF information taking 2.5% for each tail. The criterion for defining related groups (clusters) was to get unidimensional groups of minimum within variance based on the SF distribution. This criterion results in defining consecutive intervals over the range of SF values, each one reflecting a fixed proportion of SF's total variance (e.g., considering 10 intervals, the within variance of each would be approximately 10% of the total variance). The first interval includes the lowest SF values; the upper limit of this interval defines the lower tail of the SF‐distribution, whereas the lower limit of the last interval defines the upper tail. These limits were used to define COP for identifying low and high SF levels (Bergman & Magnusson, 2001; Everitt et al., 2011; Ramezani‐Tehrani et al., 2018).

Statistical analyses were run on Stata Version 14.0 software (Stata Corporation College Station, TX, USA).

## 3 RESULTS

The analytic sample for the period of measurement corresponding to 3 months of age included 746 infants who had SF and haemoglobin determination; at 6 months, 622; at 9 months, 553 and at 12 months, 532 infants. All infants had a full‐term gestation (37–42 weeks), with an average birth weight of 3,231 g, and 52.7% were boys (Table 1). Regarding breastfeeding, 2% of the participants did not receive breast milk; during the first month of life, 31% of the infants were exclusively breastfed, and 67% received mixed milk feeding (breast milk and formula). During the three first months of life, 84% received breast milk and 83% received formula for 1 to 3 months. From 3 to 6 months of life, 76% of infants received breast milk and 83% received formula for 1 to 3 months. Ruel's index score, used to evaluate the quality of complementary feeding, increased with infants' age. At 6 months of age, the median score was 5, with an interquartile range of 4–6; at 9 months old, 7 (6–8) and 8 (7–9) at 12 months of age. At 6 months old, 76% of infants were receiving breast milk, at 9 months 58% and at 12 months of age 38% of infants were breastfeeding. Approximately 80% of infants was receiving formula or bottle‐feeding from 6 months of age. In relation to dietary diversity, at 6 months of age 75% of infants ingested between one to three food groups/week, and from 9 months all infants ingested between four to six food groups/week. Fruits and vegetables had the better score in frequency of consumption from 6 months of age (≥4 times/week). The frequency of consumption of other food groups increased with age: at 6 months, 25% consumed egg or fish or poultry; red meat and cereals, legumes and tubers three times or less/week, and 75% reported no weekly consumption of these food groups; however, at 9 and 12 months, the frequency of consumption of food groups was adequate (≥4 times/week). The percentage of infants with intake of iron‐rich food also increased with age: it was 41% at 6 months, 85% at 9 months and 88% at 12 months of age.

**TABLE 1:** Baseline characteristics of the analytic sample

Characteristic	All (*n* = 746)	Girls (*n* = 353)	Boys (*n* = 393)
*Gestational age in weeks* ^a^	39 (37–42)	39 (37–42)	39 (37–42)
Type of Delivery^b^			
Vaginal	61.8	62.6	61.1
Caesarean section	38.2	37.4	38.9
Birth weight, g^c^	3,231 ± 399	3,209 ± 377	3,250 ± 417
Birth length, cm^c^	50.3 ± 2.1	50.1 ± 2.0	50.4 ± 2.2
Anthropometric indicators, Z score^c^			
Weight for age at birth	−0.15 ± 0.8	−0.08 ± 0.8	−0.23 ± 0.8
Length for age at birth	0.38 ± 1.1	0.49 ± 1.0	0.28 ± 1.1
Mother's age^b^			
Under 20 years	11.1	11.1	11.2
20 years or more	88.9	88.9	88.8
Mother's schooling^b^			
Elementary school	9.9	9.1	10.7
Middle school	33.8	33.9	33.6
High school	39.1	39.7	38.7
College or more	17.2	17.3	17.0
*Mother's employment* ^b^			
Employed	37.7	37.4	37.9
Not employed	62.3	62.6	62.1
City of residence^b^			
Zacatecas	33.1	33.4	32.8
Colima	12.2	11.6	12.7
Merida	22.5	22.7	22.4
Mexico City	32.2	32.3	32.1
Socioeconomic level^d^ ^,^ ^b^			
1	11.6	11.3	11.9
2	33.8	32.0	35.4
3	27.2	27.8	26.7
4	20.8	21.3	20.4
5	6.6	7.6	5.6
Prophylactic supplementation schedule^b^			
Ferrous sulphate, weekly	26.4	24.9	27.7
Aminochelate, weekly	22.6	24.1	21.4
Ferrous sulphate, monthly	25.1	21.8	28.0
Aminochelate, monthly	25.9	29.2	22.9

^a^ Median (minimum and maximum).

^b^ Percentage.

^c^ Mean (±SD).

^d^ Quintiles of score obtained by Hollingshead index of socioeconomic status.

The concentration of haemoglobin by measurement period was 10.8 ± 0.7 g/dL at 3 months, 11.6 ± 0.7 g/dL at 6 months, 11.7 ± 0.8 g/dL at 9 months and 11.8 ± 0.7 g/dL at 12 months. There were no statistically significant differences by sex.

### 3.1 Concentration of SF during the first year of life

SF was inversely associated with the age of the infant: as age increased, SF concentration decreased. The highest geometric mean was found at 3 months (101 μg/L, 95% CI: 96–107), with an important reduction at 6 months (39 μg/L, 95% CI: 37–42), and a continued decrease at 9 months (26 μg/L, 95% CI: 24–27) and 12 months (24 μg/L, 95% CI: 23–25). The smallest decrease was observed between 9 and 12 months compared with the previous measurements.

**FIGURE 1:**
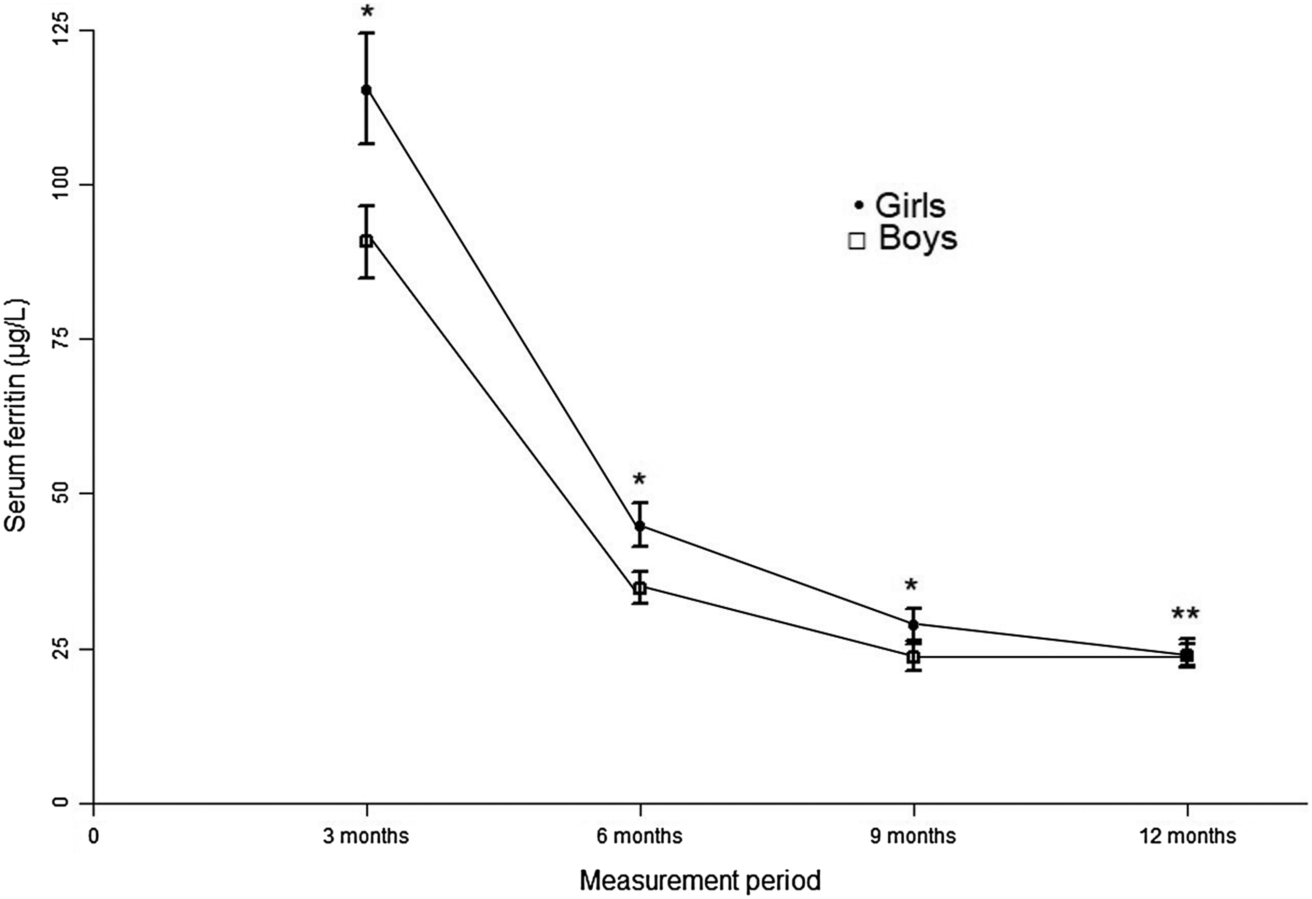
Geometric mean and 95% CI of SF by measurement period and by sex (* *p* < 0.001, ** *p* = 0.786, Student *T* test)

Girls showed systematically higher values compared to boys: geometric mean at 3 months was 115 μg/L (95% CI: 106–124) versus 91 μg/L (95% CI: 85–96), *p* < 0.001; at 6 months, 45 μg/L (95% CI: 41–48) versus 35 μg/L (95% CI: 32–37), *p* < 0.001 and at 9 months, 29 μg/L (95% CI: 26–31) versus 23 μg/L (95% CI: 21–26), *p* = 0.001. At 12 months, both girls and boys had similar values of 24 μg/L (95% CI: 22–26) versus 24 μg/L (95% CI: 22–26), *p* = 0.786. Figure 1 shows the differences in the concentration of ferritin (geometric mean) during the first year of life, stratified by sex. Expressing SF concentration by measurement periods as median and interquartile range, the values at 3 months were 101 μg/L (68–170), at 6 months 41 μg/L (25–63), at 9 months 25 μg/L (17–41) and at 12 months 24 μg/L (16–38). For girls, 117 μg/L (77–191), 46 μg/L (29–72), 28 μg/L (18–44) and 24 μg/L (16–40), and for boys, 91 μg/L (61–143), 37 μg/L (22–54), 23 μg/L (15–37) and 23 μg/L (16–36), at 3, 6, 9, and 12 months, respectively. The differences by sex in each measurement period are statistically significant except at 12 months of age.

No other differences were found in the concentration of SF according to gestational age, type of birth, birth weight, socioeconomic level or prophylactic iron supplementation schedule.

**FIGURE 2:**
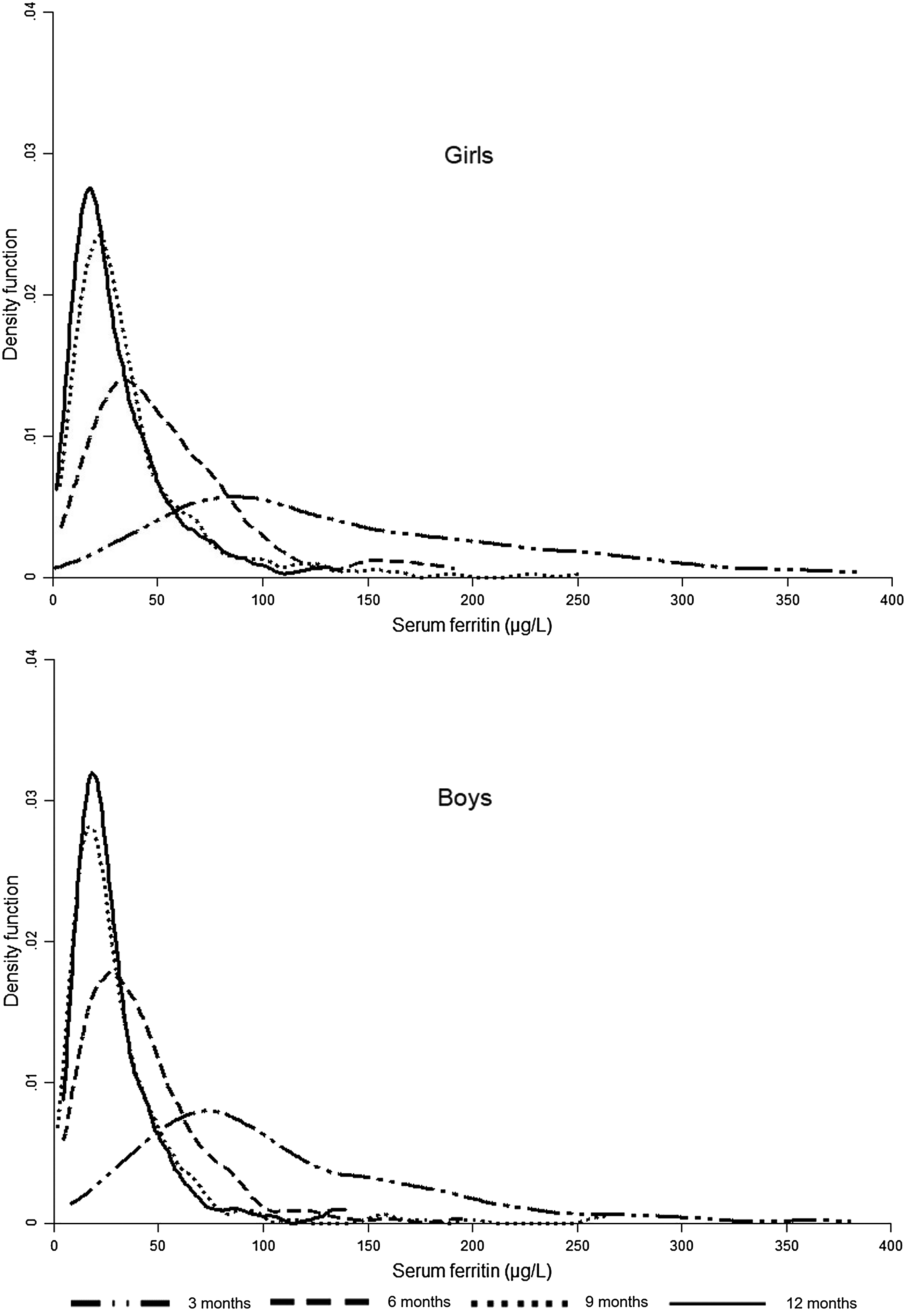
Distribution of SF by measurement period and by sex (Kernel density estimation)

The distribution of SF for each period of measurement and stratified by sex is shown in Figure 2. In all cases, the corresponding distribution is skewed to the right. The variability of the distribution of values was greater at 3 months of age. As infants grew older, this variability diminished, and the range of values shortened; at 9 and 12 months, the distributions were very similar.

### 3.2 COPs for SF concentration

The COPs for SF concentration were identified by period of measurement and sex. Table 2 shows the resulting COPs from the two analytic approaches. In the *deviation from mean*, the COPs correspond to the values of the 2.5 and 97.5 percentiles of the log SF distribution. In the *K‐means cluster analysis*, the proposed COPs were defined based on the value of the intervals' limit points. To identify low iron stores, a point‐value is taken between the upper limit of the first interval and the lower limit of the second one. Similarly, to identify high SF values, a point value is taken between the upper limit of the next‐to‐the‐last interval and the lower limit of the last interval.

**TABLE 2:** COPs for SF (μg/L) concentration to identify low iron stores and high SF level, by sex and by statistical approach

	Global COP		COP girls		COP boys	
Period of measurement	Deviation from mean^a^	K‐means cluster analysis^b^	Deviation from mean^a^	K‐means cluster analysis^b^	Deviation from mean^a^	K‐means cluster analysis^b^
3 months (*n*)	746	746	353	353	393	393
Low iron stores	25	37	26	39	25	23
High SF level	414	310	511	315	328	265
6 months (*n*)	622	622	304	304	318	318
Low iron stores	10	14	11	21	9	11
High SF level	159	140	181	165	134	115
9 months (*n*)	553	553	260	260	293	293
Low iron stores	6	10	6	10	5	10
High SF level	118	190	127	190	109	135
12 months (*n*)	532	532	256	256	276	276
Low iron stores	6	10	6	10	7	10
High SF level	94	115	104	130	86	110

^a^ *Deviation from mean* approach: COP for low iron stores: percentile 2.5, COP for high values: percentile 97.5.

^b^ *K‐means cluster analysis* approach: COP for low iron stores: value between the upper limit of the first interval and the lower limit of the second interval. COP for high SF values: value between the upper limit of the ninth interval and the lower limit of the tenth interval.

In either case, there was a noticeable reduction in the values of the COPs to identify low iron stores as age increased, with the biggest differences by sex at 3 and 6 months. Likewise, the COP at 9 months was lower than at the previous two measurements but was similar to that identified at 12 months. At 3 and 6 months, COPs for low iron stores identified by the *K‐means cluster analysis* approach were higher than those obtained by the *deviation from mean* approach, and the COPs identified by *cluster analysis* at 9 and 12 months did not differ by sex.

**FIGURE 3:**
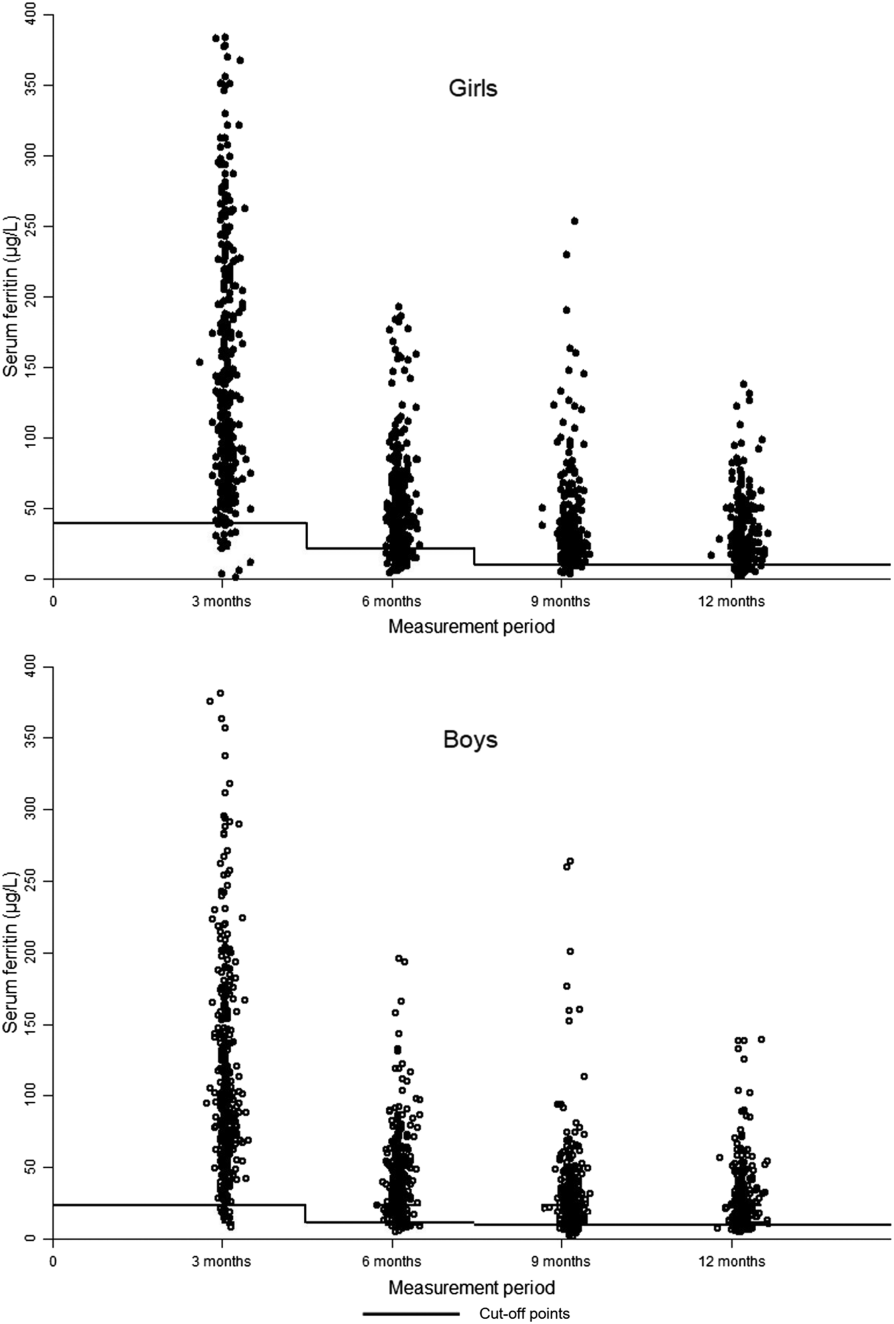
Serum ferritin and low iron stores cut‐off points, by measurement period and by sex

The values of the COPs for high SF, obtained by the *deviation from mean* approach, decreased with age, and the greatest decrease occurred between 3 and 6 months of age. This is true for the whole and sex‐stratified sample; further, COPs for girls were consistently greater than for boys. On the other hand, the behaviour of the COPs, from the *K‐means cluster analysis*, for the whole sample and by sex, shows that the values at 6 months were lower than at 3 months but those at 9 months were higher than at 6 months, and at 12 months, the values were the lowest. At 3 and 6 months, the COPs identified by the *K‐means cluster analysis* are lower than those identified by the *deviation from mean* approach, whereas at 9 and 12 months, they are higher. The COPs obtained by *K‐means cluster analysis* are consistent with the behaviour and distribution of SF observed during the first year of life (Figure 3).

## 4 DISCUSSION

Low iron stores identified by low levels of SF is the most widely used and recommended indicator to diagnose ID both individually and at a population level (Daru et al., 2017; WHO, 2007, 2011). Low SF concentration suggests deficient iron stores, whereas elevated SF levels may be indicative of an infectious or inflammatory process or suggest iron overload (Garcia‐Casal et al., 2018). Low iron stores is considered the first stage in a process that can lead to ID and IDA (Daru et al., 2017; Domellöf, et al., 2014), which is the most frequent nutritional deficiency in the world (Gupta et al., 2016; WHO, 2015). There is evidence that ID, with or without anaemia, may have long‐term consequences for neurodevelopment and behaviour, and some of these effects may be irreversible (Lozoff et al., 2006).

Physiological characteristic of the first year of life, including high iron stores at birth, greater need and use of iron due to accelerated growth and development, and progressive changes in the dietary supply and bioavailability of iron, result in large variations of iron status and SF levels. These changes have been documented by several authors. Larsson et al. (2019) and Tamura et al. (1999) reported a higher concentration of SF in umbilical cord blood in newborn girls than in boys. Domellöf, Lonnerdal, et al. (2002); Chandyo et al. (2016) and Larsson et al. (2019) observed differences in SF by sex during the first year of life, with girls showing higher SF levels. Although more research is necessary to explain the reasons for the observed sex differences, these authors suggest that possible explanations may include hormonal differences that regulate iron metabolism, or differences by sex in the foetal iron accretion or genetic factors.

The wide variations in SF require identification of dynamic COPs, with consideration of age and sex. Currently, there is no consensus about the most appropriate lower value limit of reference intervals to identify low iron stores in infants. The COPs currently recommended by WHO have limitations when applied to infants, because they were identified in older age groups and extrapolated to infancy (Domellöf, Dewey, et al., 2002; Larsson et al., 2019; Looker et al., 1997; WHO, 2007, 2011; Wu et al., 2016). It is unclear how valid they may be as population reference values (Garcia‐Casal et al., 2018).

Different authors have addressed the issue of how to identify adequate COPs for SF during infancy, resorting to different statistical methods. In variables with a normal distribution, the most common method used is the *deviation from mean* locating values 2 SD below or above the mean or at the 2.5 and 97.5 percentiles. In the case of SF, the distribution is skewed to the right, so a transformation is needed to obtain a Gaussian (normal) distribution.

Domellöf, Dewey, et al. (2002) studied a sample of iron‐replete, healthy infants who did not receive iron supplementation, showing a normal iron status at baseline, as determined by haemoglobin level and other indicators. These authors used the *deviation from mean* approach and proposed COP of 20 μg/L at 4 months, 9 μg/L at 6 months, and 5 μg/L at 9 months.

Saarinen and Siimes (1978) studied 238 healthy full‐term children, with birth weight ≥3,000 g, who had received formula since the first month of life and had determination of SF, Hb and other iron indicators. Infants who presented anaemia and/or ID were excluded. Following the *deviation from mean* approach, COPs for SF were identified as 90, 144, 87, 37, 19, 14 and 11 μg/L at ages 0.5, 1, 2, 4, 6, 9, and 12 months, respectively.

Larsson et al. (2019) used the Box‐Cox transformation of SF, presenting the lower and upper limits (reference intervals) for SF concentration by sex. The lower limits (corresponding to percentile 2.5) were 21 μg/L for girls and 16 μg/L for boys at 4 months and 13 μg/L for girls and 12 μg/L for boys at 12 months. The upper limits (percentile 97.5) were 441 and 274 μg/L at 4 months for girls and boys, respectively, and 124 and 70 μg/L at 12 months for girls and boys, respectively. In this study, infants with C reactive protein >1 mg/L were excluded.

Other approaches to identify COP for biological variables that do not show a normal distribution include Receiver Operating Curve (ROC) analysis, which may be used provided there is a gold standard of normality to find the COP from an alternative indicator (Ramezani‐Tehrani et al., 2018). When there is no gold standard or it is not appropriate for screening in epidemiologic studies, as happens with the measurement of iron in bone marrow to diagnose ID (Garcia‐Casal et al., 2018), the *K‐means cluster analyses* may be a useful method to classify groups. Cluster analysis is focused on grouping individuals in such a way that the members of the same group are the most similar among them and different from those in other groups according to a pre‐specified statistical criterion. There is no single algorithm to build the grouping; it depends on the kind of information to be had and the definition of similarity considered. Even in simple cases, the procedure for grouping is not direct; it is iterative and considers a process of trial and error. Grouping criteria is based on identifying groups with the greater homogeneity and thus minimum variance within each group (Bergman & Magnusson, 2001). *K‐means Cluster analysis* has been used for example to determine normal limits in hormones to identify hyperandrogenemia (Zhao et al., 2011) or hyperandrogenism (Ramezani‐Tehrani et al., 2018; Zhou et al., 2012) or in a study of cut‐off points to classify results of ^13^C urea breath test to identify *H. pylori* infection (Mauro et al., 2006).

In this paper, we present an analysis of a longitudinal study in which SF concentrations were measured every 3 months from the third month of age during the first year of life in a cohort of healthy full‐term infants without clinical complication during gestation and delivery, with adequate birth weight, who did not present anaemia during the first year of life and received prophylactic iron supplementation (1 mg/day of iron element) from the first month to 12 months of age, as well as advice on proper diet during the first year of life. This allowed us to document SF concentration changes during this period and estimate dynamic COPs to identify low and high SF values that reflected these trends. Similar to other studies (Domellöf, Lonnerdal, et al., 2002; Larsson et al., 2019; Miller, et al., 2006; Reinbott et al., 2016; Wu et al., 2016), we found differences in SF by age and by sex, with high values during the first semester of life, that levelled off as age increased. We used two statistical approximations to identify COP, including a *deviation from mean* approach after a logarithmic transformation of SF and one based on *K‐means cluster analysis*.

The COPs identified with *deviation from mean* approach in our study were similar to those obtained with the same method by Domellöf, Dewey, et al. (2002) and Wu et al. (2016). However, there was large variability and a right‐skewed distribution of SF in the different periods of measurement during the first year of life, and the assumption of normality of SF distribution or its logarithmic transformation was not met. The *K‐means cluster analysis* is an alternative method to identify groups, which seemed more applicable to our analysis. One advantage of this method is that it does not require distributional assumptions about the variable. Although this method is sensitive to outliers, we took care of this limitation by identifying and eliminating outliers before running the analysis. The COPs identified at the lower limit, using *K‐means cluster analysis* at 3 and 6 months of age were specific to sex and age. At 9 and 12 months of age, only one COP was identified for both sexes. The dynamic changes observed in SF during the first year of life are reflected in these COPs obtained. As expected, due to the use of different methods, these values were different than those obtained by the *deviation from mean* approach. We did not find similar studies that would allow us to compare the COPs obtained with *K‐means cluster analysis* approach. However, these COPs were similar to those obtained by Larsson using Box–Cox transformation of SF for selecting the values at 2.5 and 97.5 percentiles as low and high SF limits (Larsson et al., 2019) and to those obtained in the of Saarinen (Saarinen & Siimes, 1978).

The present study also allowed us to identify values above which SF may be considered as ‘high’ in infants. These values may not be necessarily associated with risk; they may just reflect the high end of the distribution. According to a systematic review to determine the diagnostic precision of COP of SF, the values of SF should not be used in isolation to detect iron overload (Garcia‐Casal et al., 2018). Siddappa et al. (2007) indicate that values of SF in the umbilical cord >370 μg/L are rarely found in the literature and it is most probable that they belong to neonates with the presence of inflammation or iron overload. However, information about this topic is also scarce in infants.

Our study has some limitations. (1) With respect to the analytical quality of the ferritin determination, reference materials and external quality control program were not used to evaluate the accuracy and precision of SF determinations during the execution of the assays. The determination of SF has challenges due to the presence of analytical bias and imprecision, and there is currently no reference‐method procedure for the determination of SF. However, there are reference materials (International Standards as WHO 94/572 recombinant L‐ferritin standard) that are used by commercial manufacturers of kits for the determination of SF. In clinic and research laboratories, these reference materials should also be used, in addition to proficiency testing to achieve traceability and harmonization of laboratory results. High analytical variability of SF measurements has been reported across laboratories (CV higher than 15%), increasing the rate of misclassification in clinical and epidemiological studies (Hoofnagle, 2017; Pfeiffer & Looker, 2017). (2) With respect to the age of determination of SF and haemoglobin levels, the first was done at 3 months of age. There was no blood sampling on the first month of age; at the time, the infants were included in the study. Although we recognize the limitation of not documenting the baseline iron status, we are reasonably assured that this sample of clinically healthy newborns born at term and with adequate birth weight (as determined by inclusion criteria) presented a normal iron status in the first month of life. (3) Lastly, acute phase proteins, such as C‐reactive protein or α‐1 acid glycoprotein, which can help identify an inflammatory process, were not measured. Therefore, we could not eliminate from the analysis cases where SF concentration may be altered due to the presence of an inflammatory process (Gupta et al., 2016; Larsson et al., 2019). However, we took care to reschedule the blood draw in any case where the infant showed clinical signs of infection, as well as avoiding sampling when the infant had been vaccinated in the same week as the appointment to take the blood sample. Also, infants who developed anaemia at any time during follow‐up were eliminated from the analysis. A high percentage (over 80%) of infants who had anaemia were identified as presenting anaemia not due to ID, because their SF values were above the COP identified to denote low iron stores. In these cases, anaemia possibly was related to subclinical infectious diseases, common in this age group. Other studies, in both developed and developing countries, have reported a high frequency of anaemia not related to ID in children. Petry et al. (2016), in a systematic review of national surveys, estimated that in preschool children, the proportion of anaemia associated with ID was 25% (95% CI: 18–32%). Gupta et al. (2016) reported that in the United States, approximately 28% of children who were anaemic were ID. A similar result was found in Mexican infants in 2007: 19.4% of anaemia was related to ID in infants younger than 6  months and 35.7% in infants of 6 to 11 months of age (Duque et al., 2007).

The main strength of this study was the identification of changes in SF distribution and values by sex and age throughout the first year of life in a cohort of healthy Mexican infants. These changes were reflected in the proposed SF COPs specific by sex and age to detect low iron stores. We propose that these COPs may allow us to better assess iron stores in an age group highly susceptible to developing ID and over a period where wide variations in iron status indicators are found. It is important to underscore that a COP may have different implications when applied in an epidemiological setting (such as derived from this study) or a clinical setting (which escapes the purpose of our study). In the first case, identifying applicable COPs during infancy will allow more adequate classification of population groups, to assess burden of disease or to evaluate effectiveness of interventions. In the second case, low iron stores may or may not correlate with functional outcomes, such as psychomotor development, growth, or frequency and duration of infectious diseases. These correlates are relevant if reference intervals are to guide clinical decisions (Ozarda et al., 2018), but in this study, we did not attempt to evaluate these outcomes.

To confirm the COPs proposed in this study, more studies are required for their validation, including studying populations with different genetic and ethnic backgrounds to the one presented here. It is recommended that these studies include, in addition to SF, the determination of acute‐phase proteins that allow for adjustment of iron indicators for inflammation.

## CONFLICTS OF INTEREST

The authors declare no conflict of interest.

## CONTRIBUTIONS

X.D., B.T.V., S.F.H. and H.M. designed the research study; S.F.Hdez., G.M., M.G.U, B.T., E.I.E.I, M.K.K., T.C.H., E.I.S.H., C.M.E., A.P. and E.M. performed the research; A.P.A. and B.T.V analysed the data; A.P.A. and X.D. wrote the original draft; H.M., S.F.H. and B.T.V. reviewed and edited the manuscript; S.F.Hdez., E.I.S.H., A.P. and C.M.E. coordinated and supervised the study in Mexico City, Zacatecas, Colima, and Merida, respectively. All authors have read and agreed to the published version of the manuscript.

## Data Availability

The data support the findings of this study are available from the corresponding author upon reasonable request.
